# Quantitative Characterization of α-Synuclein Aggregation in Living Cells through Automated Microfluidics Feedback Control

**DOI:** 10.1016/j.celrep.2019.03.081

**Published:** 2019-04-16

**Authors:** Giansimone Perrino, Cathal Wilson, Marco Santorelli, Diego di Bernardo

**Affiliations:** 1 Telethon Institute of Genetics and Medicine (TIGEM), Via Campi Flegrei 34, 80078 Pozzuoli (NA), Italy; 2 Department of Chemical, Materials and Industrial Production Engineering, University of Naples Federico II, Piazzale Tecchio 80, 80125 Naples, Italy

**Keywords:** bioengineering, microfluidics, feedback control, gene expression, synuclein, aggregation

## Abstract

Aggregation of α-synuclein and formation of inclusions are hallmarks of Parkinson’s disease (PD). Aggregate formation is affected by cellular environment, but it has been studied almost exclusively in cell-free systems. We quantitatively analyzed α-synuclein inclusion formation and clearance in a yeast cell model of PD expressing either wild-type (WT) α-synuclein or the disease-associated A53T mutant from the galactose (Gal)-inducible promoter. A computer-controlled microfluidics device regulated α-synuclein in cells by means of closed-loop feedback control. We demonstrated that inclusion formation is strictly concentration dependent and that the aggregation threshold of the A53T mutant is about half of the WT α-synuclein (56%). We chemically modulated the proteasomal and autophagic pathways and demonstrated that autophagy is the main determinant of A53T α-synuclein inclusions’ clearance. In addition to proposing a technology to overcome current limitations in dynamically regulating protein expression levels, our results contribute to the biology of PD and have relevance for therapeutic applications.

## Highlights

• *In silico* feedback control enables regulation of α-synuclein expression in yeast
• α-Synuclein inclusion formation is strictly concentration, but not time, dependent
• The aggregation threshold of the α-synuclein A53T mutant is 56% of the wild-type
• Autophagy induction speeds up inclusion clearance in the A53T α-synuclein strain

## Graphical Abstract

**Figure:**
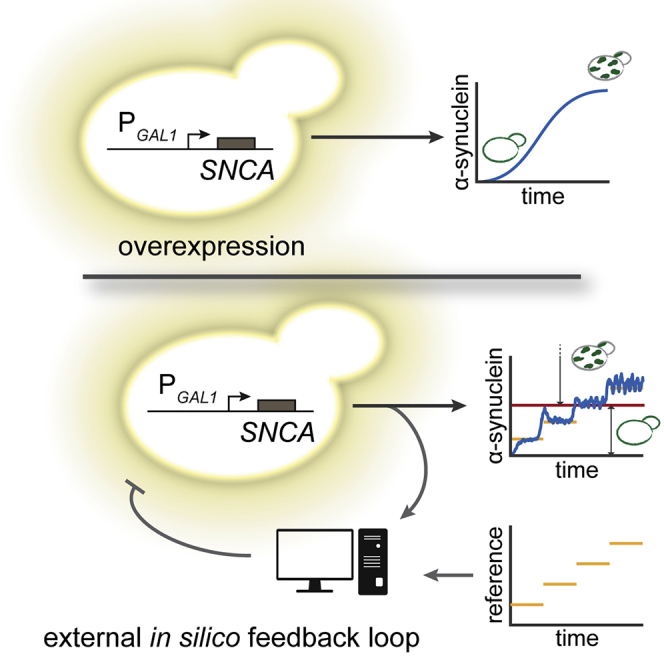

## Teaser

Perrino et al. quantitatively measured α-synuclein inclusion formation and clearance in a yeast cell model of Parkinson’s disease. A computer-controlled microfluidics device precisely regulated α-synuclein levels in cells by means of closed-loop feedback control. Inclusion formation was found to be concentration dependent, whereas inclusion clearance was mediated by autophagy.

## Introduction

α-Synuclein, encoded by the *SNCA* gene, is a small (14.5 kDa), intrinsically disordered protein expressed abundantly in a healthy brain. The precise physiological functions of α-synuclein remain poorly understood (Fusco et al., 2016), although recent findings point to a role in vesicle trafficking and synaptic physiology (Wislet-Gendebien et al., 2006, Auluck et al., 2010). In the human brain, an abnormal increase of α-synuclein expression levels may result in the aggregation of the protein into large complexes and amyloidogenic fibrils with the formation of intraneuronal proteinaceous inclusions known as Lewy bodies (Goedert et al., 2013), linked to the Parkinson’s disease (PD) pathogenesis (Goedert et al., 2017). Although the matter is still the subject of debate, it is thought that inclusions in the cell are generated by the impairment of degradative pathways and activation of the protein quality-control system (Ingelsson, 2016). The mechanisms underlying the formation of protein aggregates seem to be concentration dependent (Singleton et al., 2003). Indeed, either duplication or triplication of the wild-type (WT) α-synuclein gene locus is sufficient to cause familial PD (Singleton et al., 2003, Farrer et al., 2004, Ibáñez et al., 2004). Moreover, missense mutations in the *SNCA* gene cause early-onset (A53T, E64K, A30P, G51D, and A53E) and late-onset (H50Q) forms of PD (Polymeropoulos et al., 1997, Krüger et al., 1998, Zarranz et al., 2004, Appel-Cresswell et al., 2013, Proukakis et al., 2013, Kiely et al., 2013, Pasanen et al., 2014, Martikainen et al., 2015).

Cell-free, cellular, and animal models of PD have been developed to study the formation of inclusions (Visanji et al., 2016, Koprich et al., 2017, Lázaro et al., 2017). Pioneering studies have dissected aggregate and fibril formation in cell-free systems using purified α-synuclein protein (Giasson et al., 1999, Conway et al., 1998). These earlier *in vitro* studies were semiquantitative in that they did not quantify threshold concentrations for aggregation nor the difference between WT and mutant α-synuclein proteins. Subsequent *in vitro* studies, building on these previous works, have now precisely quantified the molecular steps of α-synuclein fibril formation and rate constants of associated reactions, thus greatly contributing to current understanding of α-synuclein pathobiology (Giehm et al., 2011, Cohen et al., 2011, Cohen et al., 2012, Buell et al., 2014, Garcia et al., 2014, Lorenzen et al., 2014, Galvagnion et al., 2015, Galvagnion et al., 2016, Flagmeier et al., 2016, Iljina et al., 2016). These *in vitro* studies have also shown that α-synuclein aggregation kinetics are strongly affected by the presence of lipid vesicles, thus highlighting the importance of studying such processes in whole cells, because the cellular environment is much more complex than the commonly used *in vitro* conditions (Flagmeier et al., 2016, Galvagnion et al., 2015).

Biological processes involved in α-synuclein inclusion formation and clearance are well conserved across evolution, hence yeast can be used to elucidate the molecular basis of the human disease and to screen for therapeutic drugs (Menezes et al., 2015, Schneider et al., 2018). Since its inception (Outeiro and Lindquist, 2003), the yeast PD model with heterologous expression of α-synuclein has been successfully used not only to study molecular mechanisms of the PD but also for high-throughput drug and genetic screenings (Zabrocki et al., 2008, Menezes et al., 2015, Chen et al., 2017). In this model, α-synuclein is expressed from the galactose-inducible promoter, and protein inclusions form with ensuing growth defects and cell death (Outeiro and Lindquist, 2003, Cooper et al., 2006, Petroi et al., 2012).

The main limitation of the yeast PD model is that despite forming α-synuclein cytoplasmic inclusions (Outeiro and Lindquist, 2003, Cooper et al., 2006), which are also found in human neurons (Giasson et al., 1999, Cooper et al., 2006), these are not comprised of insoluble α-synuclein amyloid fibrils as found in Lewy bodies (Soper et al., 2008). Rather, inclusions in yeast consist of clusters of vesicles that contain α-synuclein monomers, as well as α-synuclein aggregates formed by large oligomeric species (Soper et al., 2008, Sancenon et al., 2012, Tenreiro et al., 2014, Menezes et al., 2015). Interestingly, soluble oligomers of α-synuclein are also enriched in PD patients, and they likely represent an early aggregated form of the protein that over time transitions to much larger, insoluble aggregates and amyloid fibrils (Sharon et al., 2003).

Because α-synuclein controls vesicular dynamics and recycling in neurons, its basic functions seem to be maintained when the protein is expressed in yeast, thus making yeast a relevant model to study the biology and pathobiology of α-synuclein (Zabrocki et al., 2008).

So far, the process of α-synuclein aggregation and inclusion formation *in vivo* in the yeast PD model has been characterized only qualitatively in terms of the number of integrated genome copies because of technological limitations (Menezes et al., 2015, Outeiro and Lindquist, 2003, Popova et al., 2015). Hence, the most in-depth characterization currently available is that three copies of WT α-synuclein, versus two copies of A53T α-synuclein, are needed to observe α-synuclein inclusions in the yeast PD model (Petroi et al., 2012).

Here, we overcame current technical limitations enabling characterization of the yeast PD model quantitatively by dynamically regulating α-synuclein protein expression over time in the very same cell population, that is, without the need of using strains with different numbers of genomic integrations of α-synuclein. In so doing, we demonstrated that α-synuclein inclusion formation in yeast is strictly concentration dependent, but not time dependent. We precisely measured both the WT and disease-associated A53T α-synuclein protein aggregation thresholds, we studied the effects of inclusions on cell-cycle progression, and we dissected the contributions of proteasomal and autophagic pathways on the dynamics of A53T α-synuclein inclusions’ clearance.

In addition to contributing to the biology of PD, our results have relevance for therapeutic applications because the yeast PD model is extensively used in large-scale screening of therapeutic small molecules and modifier genes (Cooper et al., 2006, Chen et al., 2017).

## Results

### Construction and Characterization of Yeast Strains Expressing WT or Mutant (A53T) Human α-Synuclein from the Galactose-Inducible Promoter

**Figure 1:**
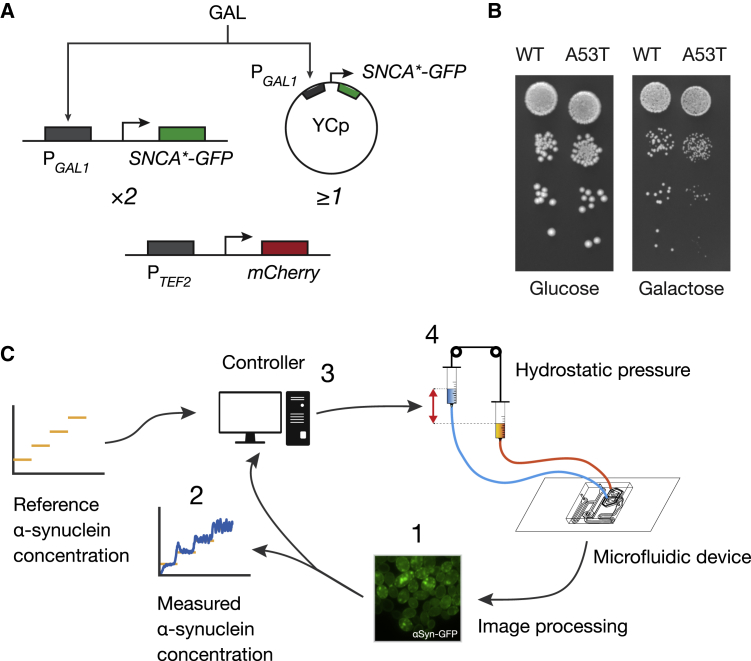
Construction and Characterization of Yeast Strains Expressing Wild-Type or Mutant (A53T) Human α-Synuclein from the Galactose-Inducible Promoter (A) Schematic of the constructs to express either the human α-synuclein gene (*SNCA*) or the disease-associated A53T mutant in yeast. α-Synuclein is fused to a GFP reporter under the control of the galactose-inducible promoter P_*GAL1*_. In each strain (WT or A53T), two copies of the construct were genomically integrated and at least one copy was expressed from a yeast centromeric plasmid (YCp) (STAR Methods). A red fluorescent reporter (mCherry protein) under the control of the constitutive promoter P_*TEF2*_ was also integrated in both yeast strains. (B) Ten-fold serial dilutions of yeast strains expressing either WT and A53T in log-phase cultures were spotted on synthetic complete drop-out media supplemented with either glucose (uninduced) or galactose (induced) to assess cell viability upon α-synuclein induction (STAR Methods). (C) Schematic illustration of the integrated experimental platform for automated microfluidics feedback control of α-synuclein in yeast cells.

**Figure 2:**
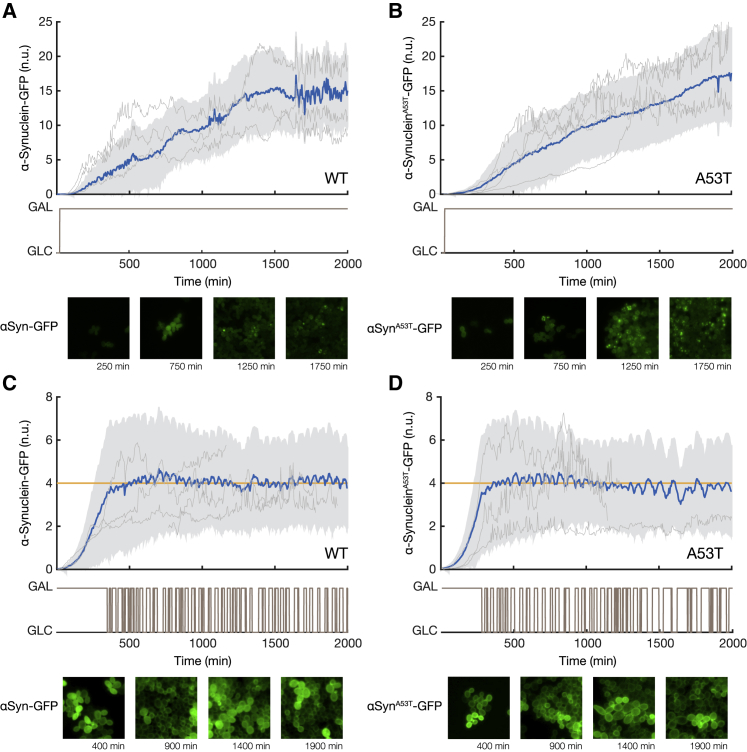
Automated Microfluidics Feedback Control Enables Precise Regulation of α-Synuclein Expression over Time in Both WT α-Synuclein Strain and A53T Mutant Strain α-Synuclein-GFP fluorescence was quantified in each cell and normalized to the red fluorescence (mCherry protein) with a custom-made image processing algorithm (STAR Methods). Light gray lines show representative examples of single-cell traces (STAR Methods). Single-cell traces may start or end at different times, because of cells being born and cells being pushed out of the field of view. (A and B) Population-averaged α-synuclein-GFP fluorescence (blue with SD across cells in gray) for the WT α-synuclein strain (A) and the A53T mutant α-synuclein strain (B) grown in the microfluidics device in the presence of galactose. Examples of images below each time course show WT and A53T mutant α-synuclein-GFP at the indicated time points. (C and D) Population-averaged α-synuclein-GFP fluorescence (blue with SD in gray) was controlled to the reference target value (yellow) by automatically switching between glucose and galactose (brown) as computed in real time by the Model Predictive Control strategy (STAR Methods). No α-synuclein inclusions were observed over the course of the experiment in both strains. Examples of images below each time course show (C) WT and (D) A53T mutant α-synuclein-GFP at the indicated time points. See also Figure S1.

We generated two *S. cerevisiae* strains expressing multiple integrated copies of either human WT α-synuclein or the mutant A53T α-synuclein fused to the GFP under the control of the GAL-inducible promoter P_*GAL1*_ (Figure 1A). Two copies were integrated in specific loci on the chromosomes, and at least one copy was expressed from a yeast centromeric plasmid (YCp) (STAR Methods). In addition, a genomically integrated red fluorescent reporter (mCherry) was constitutively expressed from the P_*TEF2*_ promoter (Figure 1A). mCherry protein fluorescence was used to normalize α-synuclein-GFP fluorescence across different experiments and strains (STAR Methods). As shown in Figure 1B, induction of α-synuclein by galactose treatment resulted in a reduced growth rate in both strains, likely caused by toxicity of protein aggregates, as previously reported (Outeiro and Lindquist, 2003, Cooper et al., 2006). Time-lapse microfluidics experiments confirmed the formation of cytoplasmic inclusions when α-synuclein was overexpressed (Figures 2A and 2B). The observed cell-to-cell variability in fluorescence levels (shadowed area in Figures 2A and 2B) is well documented and extensively studied in yeast, and is mainly caused by extrinsic noise, that is, global differences in cellular environment, including growth rate and the concentration of RNA polymerases and ribosomes (Elowitz et al., 2002, Raser and O’Shea, 2004, Rosenfeld et al., 2005, Pedraza and van Oudenaarden, 2005). We quantified cell-to-cell variability as the SD of fluorescence across cells divided by their mean for each time point (also known as coefficient of variation [CV]) as reported in Figure S1. The value of the CV is well in line with those previously observed in yeast (Volfson et al., 2006).

### Automatic Feedback Control of α-Synuclein Expression

To quantitatively study WT and mutant α-synuclein aggregation in the two strains, we made use of automated microfluidics feedback control of gene expression (Fiore et al., 2016, Menolascina et al., 2014) to precisely regulate the amount of α-synuclein produced by the cells over time, as shown in Figure 1C (STAR Methods). In brief, this automated platform performs the following steps: (1) α-synuclein-GFP expression is measured in yeast cells with a time-lapse fluorescence microscope at 5-min sampling intervals, (2) α-synuclein protein level is quantified in individual cells from fluorescent images with a custom image processing algorithm, (3) a computer implementing a controller computes the duration of the galactose pulse (ranging from 0 to 5 min) needed to minimize the difference between the target α-synuclein level and the actual population-averaged fluorescence intensity across the cells, and (4) an automated set of syringes delivers galactose (or glucose) to the cell chamber in the microfluidic device. The controller implements a model predictive control (MPC) strategy to guarantee the tracking of the target reference α-synuclein concentrations at the cell population level (Perrino et al., 2016) (STAR Methods). We first applied this control strategy to regulate α-synuclein expression at a sub-aggregation level (four normalized fluorescence units) for 2,000 min in both the WT strain (Figure 2C) and the A53T mutant strain (Figure 2D). Both time-lapse feedback control experiments did not show any protein aggregates in the cell population, unlike cells continuously grown in galactose (Figures 2A and 2B), thus demonstrating the ability of the platform to control α-synuclein expression in the cell population.

### Inclusion Formation in Both WT and Mutant α-Synuclein Strains Is Strictly Concentration Dependent

**Figure 3:**
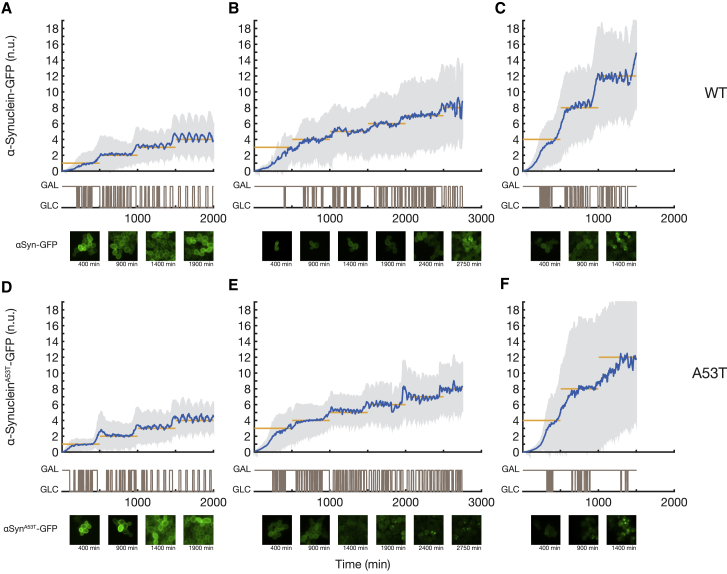
Automated Microfluidics Feedback Control of α-Synuclein Expression at Nine Increasing Levels to Observe and Quantify α-Synuclein Aggregation Thresholds in Both WT α-Synuclein Strain and A53T Mutant Strain (A–F) Six control experiments were performed to increase α-synuclein protein expression stepwise in both the WT α-synuclein strain (A–C) and mutant A53T α-synuclein strain (D–F). Population-averaged α-synuclein expression (blue with SD in gray) was tightly regulated to the target expression levels (yellow) by automatically switching between galactose and glucose (brown) as directed by the controller (STAR Methods). α-Synuclein-GFP fluorescence in single cells is normalized to mCherry fluorescence within each cell (STAR Methods) to enable comparison across cells, strains, and experiments. Examples of images below each time course show WT and A53T mutant α-synuclein-GFP at the indicated time points (A–F). Prior to the formation of inclusions, α-synuclein-GFP is mainly on the membrane, whereas inclusions appear as bright cytoplasmic spots. See also Videos S1 and S2.

We regulated α-synuclein expression to nine increasing levels in both WT and A53T mutant strains in a stepwise fashion, as shown in Figure 3. Each level was maintained for 500 min. At low expression levels (α-synuclein-GFP fluorescence ≤ 4 normalized units), both WT and A53T mutant α-synuclein proteins were localized to the cell membrane (Figures 3A and 3D). At higher concentrations, WT and A53T α-synuclein proteins began aggregating in some cells, with inclusions first appearing close to the plasma membrane (Figures 3C and 3F; Videos S1 and S2), in agreement with previous observations (Lee et al., 2002, Galvagnion et al., 2015, Popova et al., 2015).

Video S1. Controlling the Average WT α-Synuclein at High Concentrations, Related to Figure 3C

Video S2. Controlling the Average A53T α-Synuclein at High Concentrations, Related to Figure 3F

**Figure 4:**
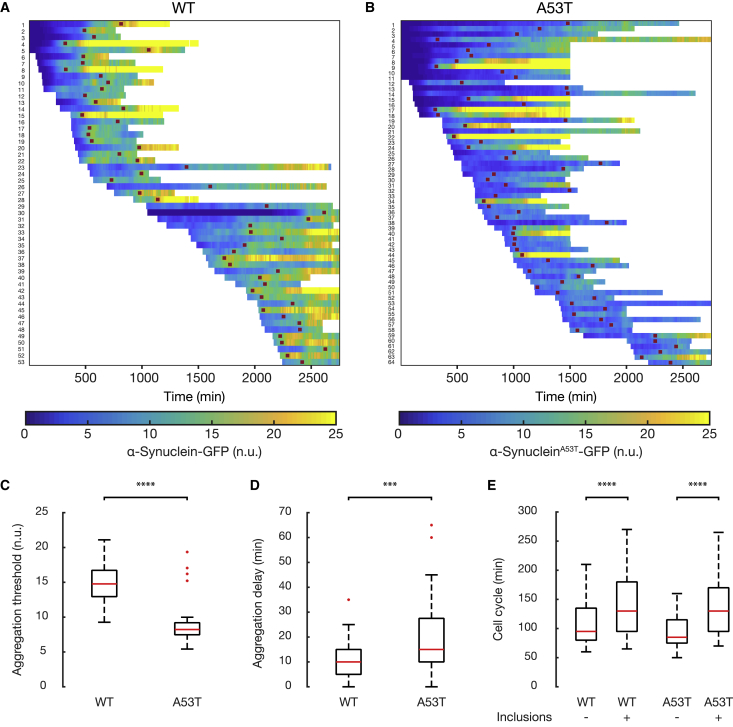
Single-Cell Time Course of α-Synuclein Expression Reveals Specific Aggregation Thresholds for Both the Wild-Type and Disease-Associated Mutant Forms α-Synuclein-GFP fluorescence was quantified in each cell and normalized to the red fluorescence (mCherry protein) with a custom-made image processing algorithm (STAR Methods). ^∗∗∗^p < 0.001, ^∗∗∗∗^p < 0.0001, Student’s t test. (A and B) Heatmap representation of single-cell α-synuclein-GFP fluorescence for the WT strain (A) and A53T strain (B) during the control experiments reported in Figure 3, where α-synuclein-GFP increases in a stepwise fashion. Each row represents a single cell and each column a 5-min time interval. Single-cell time-lapse imaging was used to detect the aggregation time point (red dot in each row), defined as the moment at which α-synuclein inclusions become visible at the cell membrane. Only cells that formed inclusions are shown. Some cells did not show any inclusions during their lifespan because they did not reach the aggregation threshold. Of the cells that reach the aggregation threshold, the majority (83% for WT and 86% for A53T) exhibited inclusions. Single-cell traces may start or end at different times, because of cells being born and cells being pushed out of the field of view. (C) Distribution of the α-synuclein fluorescence at the aggregation time point across single cells in the WT (n = 53) and A53T (n = 64) α-synuclein strains. The aggregation threshold of the WT strain is significantly higher than the A53T strain (Student’s t test, p = 1.06 × 10^−23^). (D) Distribution of the α-synuclein aggregation delay across single cells in the WT (n = 53) and A53T (n = 64) α-synuclein strains. Aggregation delay is defined as the time interval prior to the aggregation time point during which the α-synuclein-GFP fluorescence level was within 1 normalized unit from the aggregation threshold. The average aggregation delay of the A53T mutant strain is only a few minutes higher than the WT strain (Student’s t test, p = 0.0003). (E) Distributions of the cell-cycle duration across single cells before and after formation of α-synuclein inclusions in both the WT (n = 53) and A53T (n = 64) α-synuclein strains. The cell-cycle duration after α-synuclein inclusions appear increases significantly in both WT (Student’s t test, p = 7.29 × 10^−7^) and A53T (Student’s t test, p = 1.41 × 10^−17^) strains. Outliers were removed according to the boxplot rule.

To test whether there is a precise concentration threshold above which α-synuclein inclusions occur, we selected single-cell time course profiles with a minimum duration of 500 min from the time-lapse control experiments in Figure 3 (STAR Methods). We thus obtained two single-cell datasets: one for the WT α-synuclein strain and the other for the A53T mutant α-synuclein strain. For each cell, we quantified the normalized fluorescence of α-synuclein upon inclusion formation (red dots, Figures 4A and 4B). As shown in Figure 4C, the distributions of fluorescence in the WT strain are significantly higher than in the A53T α-synuclein strain (Student’s t test, p = 1.06 × 10^−23^). In order to precisely quantify WT and A53T α-synuclein aggregation thresholds, we trained a classifier that, based solely on the measured fluorescence, assigned to each single-cell image (i.e., for each cell at each time point) a label indicating the presence or absence of inclusions in the image. When the fluorescence level is above a selected threshold, the classifier assigns the “aggregation” label, whereas when the fluorescence level is below the threshold, it assigns the “no aggregation” label. The performance of the classifier was evaluated according to its accuracy, *ACC*, defined as the rate of true predictions (STAR Methods). The fluorescence threshold that gives the most accurate prediction is of 13.1 normalized units for WT α-synuclein (*ACC* = 83.26%) and 7.4 normalized units for A53T α-synuclein (*ACC* = 85.97%). These results show that the A53T mutation causes the aggregation threshold to become about half of the WT α-synuclein threshold (56%) (STAR Methods).

Next, we asked how variable this threshold is across individual cells. To this end, we computed the CV of the fluorescence of individual cells at the time of aggregation. We found that the CV is quite small for both WT and A53T mutant α-synuclein strains (*CV*_*WT*_ = 0.18; *CV*_*A53T*_ = 0.28), and that the variability in the aggregation threshold is slightly increased by the mutation.

Finally, we estimated the aggregation delay as the time needed for inclusion formation once α-synuclein-GFP fluorescence level in the cell has reached the aggregation threshold. We found that inclusions form within 12 min after reaching the aggregation threshold in the WT strain and within 20 min in the A53T strain (Figure 4D; STAR Methods). Despite these values being statistically different (Figure 4D), they differ by only 8 min, hence this difference may be negligible in biological terms, suggesting that the A53T mutation does not affect inclusion formation dynamics *in vivo*. It could also be possible that this difference is caused by the higher concentration of the WT α-synuclein needed for inclusion formation with respect to the mutant form (Figure 4C), resulting in faster protein aggregation (Galvagnion et al., 2015).

Our results support the conclusion that α-synuclein aggregation and inclusion formation in live yeast cells strictly follow a phase transition-like phenomenon governed by a threshold effect, such that inclusions will form only in cells expressing the protein above the threshold. Previous studies have suggested that yeast cells stop dividing upon α-synuclein aggregation. We therefore analyzed single-cell traces to quantify cell-cycle duration before and after aggregation. We estimated an increase in cell-cycle duration for both the WT α-synuclein strain (from 110 to 145 min; Figure 4E; Student’s t test, p = 7.29 × 10^−7^) and the A53T mutant strain (96 to 140 min, Figure 4E; Student’s t test, p = 1.41 × 10^−17^). Interestingly, we observed only a few instances of cells that completely stopped dividing upon aggregation (6% for the WT α-synuclein strain and 11% for the A53T α-synuclein mutant strain). These results show that α-synuclein toxicity in our setting mainly manifests in a slowdown of cell-cycle progression, which is similar between the WT and A53T mutant strains.

### Single-Cell Feedback Control of A53T Mutant α-Synuclein to Study Inclusion Formation and Clearance

**Figure 5:**
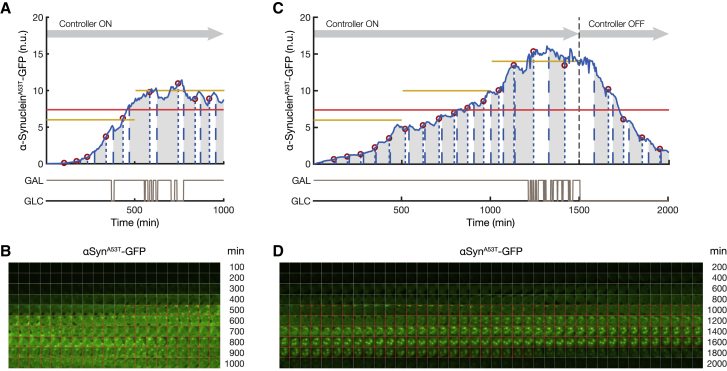
Single-Cell Automated Microfluidics Feedback Control Enables Real-Time Monitoring of Mutant A53T α-Synuclein Aggregation and Clearance Dynamics (A–D) Two single-cell control experiments are shown. α-Synuclein-GFP expression was quantified with an image processing algorithm (STAR Methods) and normalized to the red fluorescent reporter (mCherry protein; STAR Methods). Cells were tracked in real-time using a custom algorithm (STAR Methods). The budded phase of the cell cycle indicated by shaded gray areas was identified using a custom procedure (STAR Methods). Bud formation (dashed blue line) and cell division (dotted blue line) are also shown. Red circles indicate α-synuclein-GFP fluorescence at cell divisions. (A) Automatic feedback control of A53T α-synuclein-GFP expression (blue) in a single cell at two different levels (6 and 10 in yellow) below and above the A53T aggregation threshold (7.4 in red). (B) Single-cell fluorescence images of the α-synuclein-GFP time course in (A). Images are taken at 5-min intervals. Red squares highlight images where α-synuclein-GFP fluorescence level is at or above the aggregation threshold. α-Synuclein-GFP inclusions appear only once the aggregation threshold is reached. (C) Automatic feedback control of A53T α-synuclein-GFP expression (blue) in a single cell at three different levels (6, 10, and 14 in yellow) below and above the A53T aggregation threshold (7.4 in red). To investigate clearance of α-synuclein inclusions, at the end of the control experiment (1,500 min), glucose was provided to the cells, thus inhibiting A53T α-synuclein-GFP expression. (D) Single-cell fluorescence images of the α-synuclein-GFP time course in (C). Red squares highlight images where α-synuclein-GFP fluorescence level is at or above the aggregation threshold. α-Synuclein-GFP inclusions appear once the aggregation threshold is reached. Upon glucose treatment (1,500 min), α-synuclein-GFP expression decreases (from 1,500 to 2,000 min), and inclusions are cleared. See also Videos S3 and S4.

Experiments performed so far were aimed at controlling the average expression of α-synuclein across the cell population. We next regulated α-synuclein expression at the single-cell level in order to observe inclusion formation and clearance in the same cell. We first performed a time-lapse control experiment to regulate A53T α-synuclein expression at two different levels: 500 min at 6 normalized units (below the A53T mutant aggregation threshold of 7.4) and 500 min at 10 normalized units (above the aggregation threshold) (Figures 5A and 5B; Video S3). At the beginning of the control experiment, we randomly selected one cell to be controlled. Single-cell α-synuclein concentration was maintained close to the reference target levels by the automated microfluidics control platform, as shown in Figures 5A and 5B (STAR Methods). As expected, no inclusions were present when the expression level was kept close to but under the threshold level (Figure 5B). Inclusions instead became apparent when the level was increased to 10 normalized units (n.u.) (Figure 5B). Concomitantly, we observed an increase in cell-cycle duration (Figure 5A; Video S3) in agreement with the results obtained when controlling the whole population (Figure 4E). We then performed another single-cell control experiment (Figure 5C; Video S4), this time regulating A53T α-synuclein expression at three different levels: 500 min at 6 n.u., 500 min at 10 n.u., and 500 min at 14 n.u. Single-cell time-lapse imaging confirmed that α-synuclein did not form inclusions when expressed below the threshold (Figure 5D) and that the cell was dividing normally (Figure 5C; Video S4). As expected, inclusions appeared soon after the threshold was reached, together with an increase in cell-cycle duration (Figure 5C; Video S4). Interestingly, during the first 1,200 min in Figure 5C, before the cell cycle starts to slow down, a small drop in fluorescence (on average 2.86%) can be observed after each cell division. Because the Gal promoter driving α-synuclein transcription is not cell cycle dependent and because proteins are constantly produced in yeast during the cell cycle (Polymenis and Aramayo, 2015), this small drop may be the result of an α-synuclein-GFP dilution caused by volume growth during the cell cycle.

Video S3. Controlling the A53T α-Synuclein at Single-Cell Level: Two Reference Levels (6 and 10 Normalized Units), Related to Figures 5A and 5B

Video S4. Controlling the A53T α-Synuclein at Single-Cell Level: Three Reference Levels (6, 10, and 14 Normalized Units), Related to Figures 5C and 5D

To investigate the dynamics of inclusion clearance, we repressed A53T α-synuclein expression at time 1,500 min by providing glucose to the cell for an additional 500 min (Figure 5C). Interestingly, fluorescence intensity remained constant for 130 min from the removal of galactose (black dashed line in Figure 5C); afterward the cell cycle restarted and its duration returned close to pre-aggregation values despite inclusions still being present. Inclusions disappeared completely 495 min following galactose removal (Figure 5D; Video S4).

### Quantification of Autophagic and Proteasomal Contributions to the Clearance of Mutant A53T α-Synuclein

To further investigate the dynamics of A53T α-synuclein inclusions’ clearance and to dissect the relative contributions of proteasomal and autophagic pathways, we performed a series of time-lapse microfluidics experiments chemically modulating the proteasomal and autophagic pathways. Indeed, α-synuclein protein can be degraded both by the proteasome and by autophagy (Webb et al., 2003), but the latter has been proposed to be the main driver in clearing WT α-synuclein inclusions in the yeast PD model (Petroi et al., 2012, Popova et al., 2015). Here, we tested the effects of: (1) rapamycin, an autophagy inducer (Kamada et al., 2004); (2) PMSF, an autophagy inhibitor (Takeshige et al., 1992); and (3) MG132, a proteasome inhibitor (Lee and Goldberg, 1998). In order for these compounds to accumulate within yeast cells, we modified the mutant A53T strain by deleting the Pleiotropic Drug Resistance gene *PDR5* encoding an efflux transporter (Ernst et al., 2005).

**Figure 6:**
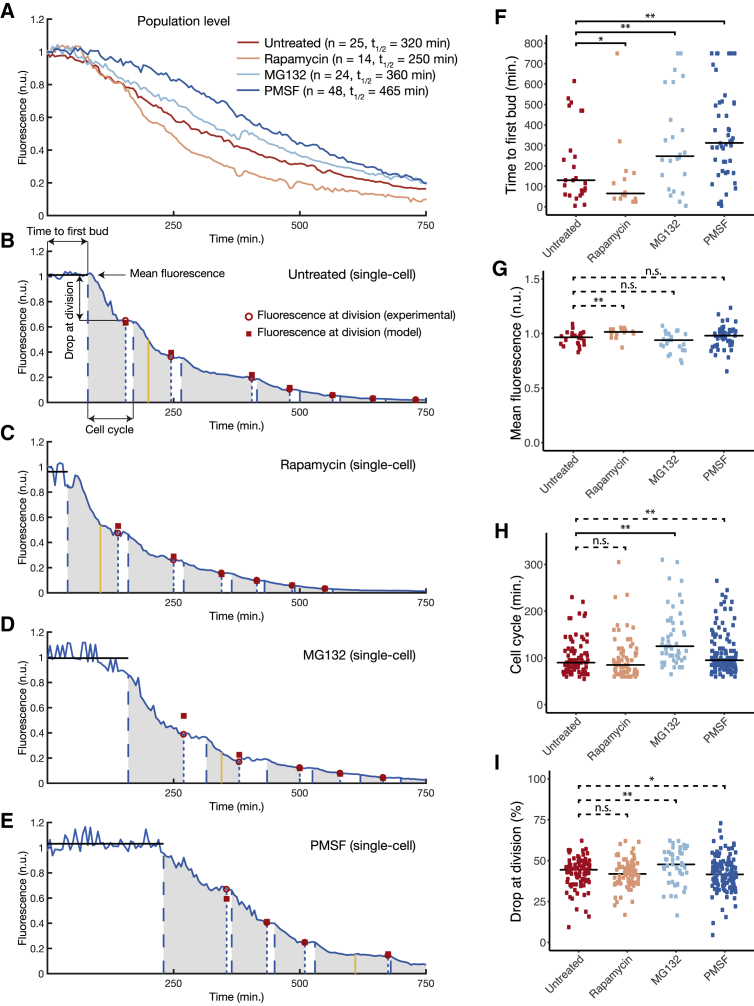
Quantification of Autophagic and Proteasomal Contributions to the Clearance of Mutant A53T α-Synuclein We tested the effects of three compounds: (1) rapamycin (100 nM), an autophagy inducer; (2) PMSF (1 mM), an autophagy inhibitor; and (3) MG132 (50 μM), a proteasome inhibitor, in the mutant A53T α-synuclein strain deleted for the Pleiotropic Drug Resistance gene *PDR5* encoding an efflux transporter to enable accumulation of the small molecules (STAR Methods). Cells were grown overnight in the presence of galactose to induce formation of α-synuclein inclusions. At time 0 min, galactose was replaced by glucose, and α-synuclein-GFP fluorescence was quantified in individual cells in the microfluidics device using a custom-made image processing algorithm (STAR Methods). Only cells that were present from the beginning to the end of the experiment were considered. (A) Population-averaged α-synuclein-GFP fluorescence computed from single-cell traces (STAR Methods) in each of the four conditions tested. *t*_1/2_ is time necessary for α-synuclein-GFP fluorescence to become half of its initial value. (B–E) Representative single-cell time course (solid blue lines) in the Δpdr5 A53T α-synuclein yeast strain for each of the indicated conditions: untreated (B), rapamycin (C), MG132 (D), and PMSF (E). α-Synuclein-GFP fluorescence in each individual cell is normalized to the mean fluorescence during the calibration phase (STAR Methods). The time at which α-synuclein inclusions disappear is indicated by a yellow line (STAR Methods). The budded phase of the cell cycle is indicated by shaded gray areas. Bud formation (dashed blue line) and cell division (dotted blue line) are also shown. We defined four parameters as indicated in (B): time to first bud is defined as the time elapsed between the beginning of the experiment and the formation of the first bud of the cell. The mean fluorescence (solid black lines) is defined as the average fluorescence from the beginning of the experiment until the time to first bud. The drop of fluorescence at division is defined as the percentage decrease in fluorescence during the budded phase. The cell-cycle duration is defined as the time between two consecutive budding events. Experimental fluorescence at division (red circles) and model-predicted fluorescence, assuming a drop in fluorescence of 38% at division (Jonas et al., 2018) caused by dilution (red squares), are also shown. (F–I) Distribution of time to first bud (F), mean fluorescence (G), duration of cell cycle (H), and drop at division (I) across single cells in the different conditions: untreated (n = 25), rapamycin (n = 14), MG132 (n = 24), and PMSF (n = 48). Solid black lines are the medians in each condition. Horizontal square brackets represent statistically significant pairwise comparisons with median values between conditions changing by at least 10%. Dashed horizontal square brackets represent pairwise comparison with median values between conditions changing less than 10%. ^∗^p ≤ 0.1, ^∗∗^p ≤ 0.05; Conover-Iman test of multiple comparisons using rank sums. The number of points in (H) and (I) is higher because each cell undergoes multiple divisions during the experiment; each point refers to a cell cycle in untreated (n = 91), rapamycin (n = 73), MG132 (n = 49), and PMSF (n = 137) condition. n.s., not significant. See also Figures S2 and S3 and Video S5.

We analyzed A53T α-synuclein inclusions’ clearance both at the population and single-cell level in the mutant Δpdr5 A53T strain upon treatment with the three compounds, as shown in Figure 6. We grew cells overnight in the presence of galactose in the microfluidics device to induce formation of α-synuclein inclusions; at time 0 min, galactose was replaced by glucose and fluorescence was quantified in individual cells (STAR Methods).

Figure 6A reports the population-averaged fluorescence computed from single-cell traces across the indicated conditions. Enhancement of autophagy by rapamycin (yellow line) accelerates clearance compared with untreated control (red line), whereas inhibition of autophagy by PMSF slows it down (dark blue line). The proteasome inhibitor MG132 also slowed clearance dynamics, but to a lesser extent than PMSF (light blue line). These results clearly support the role of autophagy as a major player in the clearance of A53T α-synuclein inclusions.

At the population level, however, it is not possible to assess what are the main determinants of the observed differences in A53T α-synuclein inclusions’ clearance dynamics. We thus analyzed single-cell traces upon the different treatment conditions (Figures 6B–6E). For each cell, we measured four parameters as depicted in Figure 6B: (1) the time to first bud (TFB), defined as the time elapsed between the beginning of the experiment and the formation of the first cell bud; (2) the mean fluorescence value during the TFB; (3) the duration of each cell cycle; and (4) the drop at division after each cell cycle, defined as the percentage decrease in fluorescence between two consecutive cell cycles. Figure 6F shows the distribution of the TFB for all the cells and across treatments. Rapamycin significantly reduced the TFB compared with untreated cells, whereas PMSF increased it, as did MG132 albeit to a lesser extent (Figure 6F). Interestingly, the mean fluorescence value during the TFB was not significantly affected by treatments and remained close to 1, which is the value of fluorescence at the beginning of the experiment (Figure 6G). Cell-cycle duration, in Figure 6H, was significantly increased by MG132 treatment only. This effect of MG132 has been previously reported; indeed, these cells exhibit morphological defects forming pseudohyphae, which are probably caused by impairment of cell-cycle proteins’ degradation (Collins et al., 2010). The drop in fluorescence between consecutive cell cycles is reported in Figure 6I and was not significantly affected by any treatment, and on average it is less than 50% independently of the treatment. This is in agreement with a dilution mechanism caused by yeast asymmetric cell division, where the mother cell is reported to shed on average 38% of its total mass to the daughter cell (Jonas et al., 2018). Indeed, a simple mathematical model that assumes a drop of 38% in fluorescence at each cell cycle recapitulates the decrease in fluorescence over time in each of the cells independently of the treatment (red squares in Figures 6B–6E; Figure S3) (STAR Methods).

This quantitative analysis shows that the clearance dynamics of α-synuclein inclusions observed at the population level (Figure 6A) largely result from the time it takes for each cell to bud following inhibition of α-synuclein expression by glucose, i.e., the TFB in Figure 6F. Interestingly, during this time no measurable decrease in fluorescence can be observed (Figure 6G), whereas inclusions are still present (Figures 6B–6D; Video S5). It has been reported that GFP is relatively stable in the vacuole, hence it may be possible that only the α-synuclein moiety of the α-synuclein-GFP fusion protein is being degraded during this time (Delorme-Axford et al., 2015). We also computed the time it takes for α-synuclein inclusions to disappear in each single cell across the different conditions and found it to be highly correlated with the TFB (Pearson’s product moment correlation coefficient r = 0.77; p < 2.2 × 10^−16^; Figure S2B). This result shows that the TFB can be used as a proxy to predict when inclusions will disappear.

Video S5. Clearance of Mutant A53T α-Synuclein for the Single-Cell Traces, Related to Figures 6B–6E

Finally, we checked whether impairment of either proteasomal or autophagic pathways affects α-synuclein protein clearance when this is expressed below its aggregation threshold and thus when no inclusions are present. We performed microfluidics control experiments in the mutant Δpdr5 A53T strain to reach and maintain an α-synuclein expression level of 4 normalized units for 500 min, which is below the aggregation threshold of mutant A53T synuclein, followed by inhibition of α-synuclein expression for an additional 500 min, as shown in Figure S3. At the population level, treatment with either rapamycin or PMSF had no measurable effects on clearance dynamics (Figure S3), demonstrating that autophagy has no role in A53T α-synuclein protein clearance when inclusions are not present. Cells treated with MG132 showed a marginal impairment of the clearance dynamics (Figure S3), which is likely driven by MG132 effects on the cell cycle and hence dilution, rather than to a direct effect of proteasome inhibition on α-synuclein. Indeed, because inclusions are not present, yeast cells continuously proliferate and hence dilution caused by cell division is the main driver of the fluorescence decay in these experiments.

## Discussion

In this work, we applied an innovative technological platform to quantitatively study the effect of α-synuclein dosage in living cells in real time. Our platform is based on the application of Control Engineering to biological systems (a field named cybergenetics), and it represents a significant advance compared with previous studies assaying α-synuclein inclusion formation and clearance. Recently, a microfluidics device has been built to generate a chemical gradient resulting in nine different concentrations of galactose (or other small molecules) to induce different α-synuclein levels in a yeast PD model, but no inclusions were observed by galactose administration alone (Fernandes et al., 2014). Previous studies have also investigated clearance of WT α-synuclein inclusions in the yeast PD model, suggesting an involvement of autophagy (Petroi et al., 2012, Popova et al., 2015). However, the phenomenon was not quantitatively investigated at the single-cell level.

Here, we performed the detailed single-cell quantitative characterization of the yeast PD model and demonstrated that formation of inclusions in both WT and A53T mutant α-synuclein yeast strains is strictly concentration dependent, in agreement with previous results obtained in cell-free models (Iljina et al., 2016). Moreover, we precisely measured the *in vivo* aggregation threshold in both strains and demonstrated that the A53T mutation causes the threshold to become about half of the WT α-synuclein threshold (Figure 4; Table S4), and that if a cell expresses the A53T protein above the threshold it will form inclusions with 86% probability (i.e., the accuracy of the classifier in Table S4).

In addition, whereas the cell cycle slows down in the presence of inclusions and eventually stops (Figure 4E), there is not a quantitative difference between WT and A53T α-synuclein, implying that the increased toxicity observed in yeast growth assays has to be attributed uniquely to the lower aggregation threshold of A53T.

We chemically modulated the proteasomal and autophagic pathways to dissect their contribution to α-synuclein inclusions’ clearance. We quantitatively characterized clearance dynamics at the population level and at the single-cell level for two different concentrations of A53T α-synuclein, both below and above the aggregation threshold. We found that: (1) enhancing autophagy speeds up A53T α-synuclein inclusions’ clearance, whereas repression of autophagy impairs it, with proteasome inhibition causing an intermediate effect; and (2) autophagy modulation has no effect on A53T α-synuclein clearance dynamics when its expression is below the aggregation threshold, and thus when inclusions are not present. By performing a detailed single-cell quantitative analysis of clearance dynamics, we demonstrated that the time it takes for the cell to bud following inhibition of A53T α-synuclein expression is the parameter most affected by autophagy modulation, whereas once cell division occurs, dilution is the main driver of inclusion clearance in all conditions tested. This means that autophagy exerts its beneficial effects in yeast before cell division occurs, enabling the cell to divide. This finding may impact the way the PD yeast model will be used for studying disease mechanisms and for screening drugs and targets. For example, the readout of most chemical and genetic screens in the yeast PD model is number of colonies formed in galactose, when inclusions are present. This phenotype can be measured only at the population level and requires considerable time to allow for colonies to become visible. We propose instead to adopt the TFB as the screening parameter to evaluate the efficacy of genetic and chemical perturbation, because it can be measured in single cells over a shorter period of time.

Our results show that the formation of inclusions in yeast is a phase-transition-like phenomenon, suggesting that inclusions form in equilibrium with α-synuclein monomers and thus should “passively” dissolve as α-synuclein concentration decreases below the aggregation threshold, unlike the insoluble amyloid aggregates found in Lewy bodies. We demonstrated that rapamycin treatment can considerably speed up inclusion clearance in yeast. This phenomenon may be explained either by assuming that inclusions in yeast are actively degraded by autophagy, or by the effect of rapamycin on translational repression, which reduces the amount of the available α-synuclein protein, or still by other indirect effects. However, when we treated cells with the vacuole protease inhibitor PMSF, inclusion clearance was considerably delayed, thus suggesting that active degradation by autophagy is the main player.

Protein aggregation plays key roles in cellular processes as wide ranging as disease initiation and progression, signaling, and evolution. Automated microfluidics feedback control represents a technology to quantitatively probe the phenomena linked to protein dosage. Moreover, we recently demonstrated that a similar microfluidics platform can be used to regulate protein expression in mammalian cells (Postiglione et al., 2018), suggesting the possibility of performing similar quantitative studies in human neuronal cell lines in the near future. Our approach can be easily extended to unravel the dynamics of other proteins prone to aggregation, such as the tau protein.

## STAR★Methods

### Key Resources Table

**Table:** 

REAGENT or RESOURCE	SOURCE	IDENTIFIER
**Chemicals, Peptides, and Recombinant Proteins**		

Rapamycin from *Streptomyces hygroscopicus*	Sigma-Aldrich	Cat#R0395-1mg
MG132	Calbiochem	Cat#474791
PMSF	Sigma-Aldrich	CAS: 329-98-6

**Deposited Data**		

Zenodo data repository	This study	https://doi.org/10.5281/zenodo.2546466

**Experimental Models: Organisms/Strains**		

Yeast strains	Table S1	Table S1

**Recombinant DNA**		

Plasmids	Table S2	Table S2

**Software and Algorithms**		

MATLAB	The MathWorks	N/A
R	The R Foundation	N/A

### Contact for Reagent and Resource Sharing

Further information and requests for resources and reagents should be directed to and will be fulfilled by the Lead Contact, Diego di Bernardo (dibernardo@tigem.it).

### Experimental Model and Subject Details

#### Yeast Strains and Growth Conditions

All *Saccharomyces cerevisiae* strains used in this study are listed in Table S1. For all experimental conditions, cells were cultured at 30°C in synthetic complete drop-out media, composed of yeast nitrogen base (0.67%w/v) with all amino acids except leucine, supplemented with either glucose (2%w/v), or raffinose (2%w/v), or galactose (2%w/v) and raffinose (2%w/v).

### Method Details

#### Plasmid Construction

All plasmids used in this study are listed in Table S2. WT and A53T α-synuclein expression cassettes were amplified from pRS304-αSynWT-GFP and pRS304-αSynA53T-GFP, respectively, (a kind gift from S. Lindquist lab) via PCR and cloned into both pYM27 (kanamycin selection) and pYM25 (hygromycin selection) between SalI and BglII restriction sites or into the centromeric plasmid YCplac111. All plasmid sequences were checked by Sanger sequencing.

#### Strain Construction

All yeast strains used are listed in Table S1. The strain constitutively expressing mCherry was derived from a strain with TEF2_pr_-mCherry::URA3 integrated into the URA3 locus (Breker et al., 2013). The αSyn expression cassettes were PCR-amplified from the pYM27 and pYM25 constructs using oligonucleotides containing sequences homologous to the 5′ and 3′ UTR of the dubious ORFs YMR082C or YFR054C, respectively. Strain yDdB001 (expressing mCherry) was transformed sequentially with the respective amplicons using standard procedures. Correct integrations were verified by PCR on extracted genomic DNA. The multiple copy WT α-synuclein strain contains two integrated WT α-synuclein expression cassettes (YMR082CΔ::GAL1p-SNCA-GFP-KanMX and YFR054CΔ::GAL1p-SNCA-GFP-HphMX) and at least one copy expressed from a yeast centromeric plasmid (YCplac111-GAL1p-SNCA-GFP-LEU2). The multiple copy A53T α-synuclein strain contains two integrated A53T α-synuclein expression cassettes (YMR082CΔ::GAL1p-sncaA53T-GFP-KanMX and YFR054CΔ::GAL1p-sncaA53T-GFP-HphMX) and at least one copy expressed from a yeast centromeric plasmid (YCplac111-GAL1p-sncaA53T-GFP-LEU2). Deletion of the Pdr5 gene was performed by amplifying the natNH2 gene (nourseothricin selection) from plasmid pRS41N using oligonucleotides containing sequences homologous to the 5′ and 3′ UTR of the Pdr5 gene, followed by transformation of the multiple copy A53T α-synuclein strain.

#### Spotting Assays

Cells from a frozen glycerol stock (−80°C) were resuspended in 1mL of growth media supplemented with glucose (2%w/v), grown in a shaking incubator at 220r.p.m. and 30°C to log phase. The cells were diluted to the concentration of $OD_{600}\;=\;0.1$ $\left(∼3\times 10^{6}cells/mL\right)$, and then ten-fold serially diluted and spotted onto synthetic complete drop-out media plates supplemented either with glucose (uninduced; 2%w/v) or galactose (induced; 2%w/v). Plates were incubated at 30°C for 3 days.

#### Microfluidics

All microfluidics experiments were performed by means of the MFD0005a device (Ferry et al., 2011). This device contains a chamber in which the yeast cells are trapped. The height of the chamber (3.5μm) allows the yeast cells to grow only in a monolayer, simplifying the image analysis process. Microfluidic devices were fabricated with a replica molding technique. The master-mold was produced using multilayer soft-lithography with SU-8 as photoresist. Before the fabrication, the master-mold was exposed to vapors of chlorotrimethylsilane (CARLO ERBA Reagents) for 10min in order to create an anti-sticking layer for polydimethylsiloxane (PDMS; Sylgard 184, Dow Corning). The PDMS was poured on the top of the master-mold with a 1:10 ratio (curing agent to base; w/w) and then cured at 80°C for 2hrs. Next, the PDMS layer was cut and peeled from the master-mold. The inlet ports of the PDMS device were pierced with a micro-puncher (0.5mm; World Precision Instruments), and then the PDMS device was washed overnight in isopropyl alcohol. A cover glass (thickness no. 1.5; Marienfeld-Superior) was first cleaned in acetone, then washed in ddH2O, and finally cleaned in isopropyl alcohol. The PDMS device and the cover glass were left overnight to dry, and then bonded together irreversibly by undergoing a plasma treatment for 1min in a plasma cleaner machine (ZEPTO version B; Diener electronic). The bonded device was baked for 2hrs at 80°C, and then stored at 4°C until use. The fluid that reaches the chamber of the microfluidic device is a mixture of the growth media coming from the two inlet ports of the microfluidic device. The blending of the growth media depends on the relative pressure between the two fluids at the inlet ports. In order to change the relative pressure, we devised an automated actuation system that varies the relative height of the two syringes filled with the inducing/uninducing media. The actuation system relies on two custom vertically mounted linear actuators, that are conceived to move independently. Each linear actuator comprises a stepper motor that realizes the motion of the syringe through a timing belt and two pulleys. A custom MATLAB script pilots the stepper motors in order to realize the galactose pulse according to the computed control input.

#### Phase Contrast and Epifluorescence Microscopy

Phase contrast and epifluorescence images were acquired at 5 min intervals at 40 × magnification (CFI Plan Fluor DLL 40 × dry objective, NA 0.75; Nikon Instruments) using an Eclipse Ti-E inverted microscope (Nikon Instruments) coupled with an EMCCD camera (iXon Ultra 897; Andor Technology) and a Perfect Focus System (Nikon Instruments). The microfluidic device was placed on the microscope stage within a 30°C incubator system. Appropriate filters were used for acquiring the green (Piston GFP; Nikon Instruments) and the red (TRITC HYQ; Nikon Instruments) fluorescence channels. Time-lapse acquisition was controlled by the NIS-Elements Advanced Research software (Nikon Instruments). Raw phase contrast and epifluorescence images were processed using custom MATLAB software. The procedures to quantify the α-synuclein expression levels among the time-lapse experiments are described below.

#### Image Processing

The procedures for quantifying the α-synuclein expression levels were performed using custom scripts in the MATLAB environment. Yeast cells were localized by processing the phase contrast images with a custom segmentation algorithm. Raw phase contrast images were enhanced by using the MATLAB function *imadjust* of the *Image Processing Toolbox*. A Gaussian low pass filter was first designed with the MATLAB function *fspecial* of the *Image Processing Toolbox*, and then applied to the enhanced images with the MATLAB function *imfilter* of the *Image Processing Toolbox*. Next, yeast cells were found in the processed phase contrast images by using the circular Hough Transform. The circular Hough Transform detects the circles in the image, and it is implemented by the MATLAB function *imfindcircles* of the *Image Processing Toolbox*. The function returns a two-column matrix containing the centroids of the yeasts found in the phase contrast images, and the radii associated to each centroid. Such information was employed to create a binary mask that defines the pixels of the fluorescence images associated to the yeast cells. Fluorescence intensities were quantified by processing the fluorescence images (green channel and red channel) with the binary masks described above and a custom MATLAB script. Average fluorescence levels were computed by integrating the signals from the selected pixels. Small rectangular regions outside the microfluidic chamber were used to eliminate the basal fluorescence levels. The basal fluorescence levels were first computed by integrating the fluorescence signals emitted by the pixels belonging to these regions, and then subtracted from the average fluorescence levels. Note that the fluorescence intensities are measured in arbitrary units. Evolution of single cells were tracked using a custom MATLAB script. The script comprises a tracking module that searches the correspondences between the objects (i.e., the centroids) detected in two consecutive phase contrast images by minimizing a cost configuration (i.e., the displacements among the centroids of two consecutive images) and a lineage analysis module that builds a genealogical tree containing the mother-daughter relationships among the yeast cells. Since the tracking module is computationally expensive to be executed in real-time, a GPU-parallel software implementation of the tracking module was developed (Rea et al., 2018).

#### Feedback Control Strategy

All control experiments were performed by means of a model predictive control (MPC) algorithm, which has been successfully applied to control gene expression and protein activation in yeast (Chait et al., 2017, Fiore et al., 2016, Milias-Argeitis et al., 2016, Uhlendorf et al., 2012). MPC is an optimization-based technique which uses a dynamical model to predict the future behavior of the controlled system in order to compute the best control input to steer the system output toward the desired reference (Camacho and Alba, 2007). The MPC algorithm was implemented as previously described unless otherwise specified (Fiore et al., 2016). In our system, at each sampling time kT, MPC computes the optimal duration d of the galactose pulse (ranging from 0 min to 5 min, assuming a period T equals to 5 min) needed to minimize a cost index (SSE, sum of the predicted squared control error) over a prediction horizon $T_{P}$ (2hrs). The control error e is defined as the difference between the desired α-synuclein level $y_{ref}$ and the measured (or predicted, as in the case of the MPC algorithm) α-synuclein level y. Optimization was performed with the MATLAB function *fmincon* of the *Optimization Toolbox*. Sequential Quadratic Programming (SQP) was used as solver method. The dynamical model used by the MPC algorithm consists of three difference linear equations:

$$x_{1}\left(k+1\right)=a_{11}\;x_{1}\left(k\right)+a_{12}\;x_{2}\left(k\right)+b_{1}\;u\left(k\right)$$

$$x_{2}\left(k+1\right)=a_{21}\;x_{1}\left(k\right)+a_{22}\;x_{2}\left(k\right)+b_{2}\;u\left(k\right)$$

$$x_{3}\left(k+1\right)=x_{3}\left(k\right)$$

and a further equation that describes the system output:

$$y\left(k\right)=c_{1}\;x_{1}\left(k\right)+c_{2}\;x_{2}\left(k\right)+x_{3}\left(k\right)$$

In the above equations, $x_{i}\in {ℜ}^{3}$represents the system state, which has no physical meaning in our model. Instead, u represents the control input, and it is mathematically described as:

$$u\left(k\right)\;=\frac{d\left(k\right)}{T}$$

Since d≤T⇒u∈[0,1]. We thus assumed that the control input is piecewise constant during the period T (zero-order hold method) (Franklin et al., 1990). Moreover, the state variable $x_{3}$ augments the order of the dynamical model in order to reduce the steady-state error (Pannocchia and Rawlings, 2003). State system is numerically estimated from the measured fluorescence data by using a Kalman filter. Model parameters used in this study are reported in Table S3. The MPC algorithm was implemented by custom scripts in the MATLAB environment.

#### Experimental Setup

For microfluidics control experiments (Figures 2, 3, and S3), cells from a frozen glycerol stock (−80°C) were resuspended in 10mL of growth medium supplemented with raffinose (2%w/v), grown overnight in a shaking incubator at 220r.p.m. and 30°C; poured in a 50mL syringe (BD), and then injected in the microfluidic device as previously described (Fiore et al., 2016). Unless otherwise specified, cells were left to settle in the chamber for 15 min with growth medium supplemented with glucose (2%w/v). Image acquisition was then launched, as well as the custom software for running the time-lapse control experiment. At the beginning of the experiment, a *region of interest* (ROI) was selected on the first phase contrast image. The ROI defines the area containing the cellular population that has to be segmented and whose fluorescence signals have to be quantified. Indeed, all custom software considers only cells inside this area. Outside the chamber another area is selected on the first phase contrast image, that defines the background to quantify the basal fluorescence levels in each fluorescence channel. For single-cell control experiments (Figure 5), the cell to be controlled was chosen randomly at the end of the initial calibration phase (15 min in growth medium supplemented with glucose). For inclusions’ clearance experiments (Figure 6), cells from a frozen glycerol stock (−80°C) were resuspended in 10mL of growth medium supplemented with raffinose (2%w/v) and galactose (2%w/v), grown overnight in a shaking incubator at 220r.p.m. and 30°C; poured in a 50mL syringe (BD), and then injected in the microfluidic device as previously described (Fiore et al., 2016). Cells were left to settle in the chamber for 10 min with growth medium supplemented with galactose (2%w/v) and raffinose (2%w/v). Image acquisition was then launched. After 10 min, galactose and raffinose were replaced by glucose (2%w/v). For all microfluidics experiments, the experimental platform was initialized as previously described (Fiore et al., 2016).

#### Treatments for Inclusions’ Clearance Experiments

Three compounds were used to study the clearance of α-synuclein inclusions: (i) rapamycin at a concentration of 100nM, (ii) phenylmethane-sulfonyl-fluoride (PMSF) at a concentration of 1mM, and (iii) MG132 (also known as carbobenzoxy-Leu-Leu-leucinal) at a concentration of 50μM. All the compounds were added to the growth medium supplemented with glucose.

### Quantification and Statistical Analysis

Statistical details are provided in the figure legends unless otherwise specified. The α-synuclein concentrations in both Figures 2 and 3 are presented as means ± standard deviations. Quantification and statistical analysis of the single-cell aggregation analysis were performed in the MATLAB environment. The statistical comparisons were made using the Student’s t test. In these analyses, a p−value<0.05 was considered to be statistically significant. Quantification and statistical analysis of the inclusions’ clearance analysis were performed in the R environment. The statistical comparisons were made using the Conover-Iman test of multiple comparisons using rank sums, available in the *conover.test* package. Biological significance is defined as a fold change greater than 10% for the median values between conditions.

#### Normalization of α-Synuclein Expression Level

Unless otherwise specified, the fluorescence of the α-synuclein-GFP in each cell was normalized by dividing the α-synuclein-GFP level measured at each time point by the mean mCherry level measured during the initial calibration phase. In the clearance analysis (Figure 6), the fluorescence of the α-synuclein-GFP in each cell was normalized by dividing the α-synuclein-GFP level measured at each time point by the mean α-synuclein-GFP level measured during the initial calibration phase of 5 min.

#### Single-Cell Analysis of Control Experiments

Single-cell data were collected from the time-lapse control experiments (Figure 3) according to the following procedure. The custom segmentation and tracking algorithm (Method Details) processed the phase contrast images to extract single-cell traces. We selected only single-cell traces longer than 500min. We then discarded the traces that did not reach a minimum expression level during the time-course (16 normalized units for WT strain and 10 normalized units for A53T mutant strain). We thus obtained 94 traces for the WT strain and 128 for the A53T mutant strain. For each cell trace, we quantified the normalized fluorescence of α-synuclein during the time-course. Next, we identified the cells that showed α-synuclein-GFP aggregates during the time-course (53 out 94 for WT strain and 64 out of 128 for the A53T). To this end, we manually inspected single-cell movies to check if the cells formed aggregates during the time-course. If so, we collected the aggregation time point $t_{A}$ defined as the time point at which the aggregates are visible at the cell membrane. We obtained two datasets, one for the WT α-synuclein and one for the A53T mutant α-synuclein. The aggregation threshold in each cell is defined as the α-synuclein-GFP normalized fluorescence level at the aggregation time-point $t_{A}$. Aggregation delay in each cell is defined as the time interval prior to the aggregation time-point $t_{A}$ during which the α-synuclein-GFP fluorescence level was within 1 normalized unit from the aggregation threshold. Cell cycle duration in each cell is defined as the time interval that occurs between two consecutive budding events. Cell cycle duration was quantified by inspecting manually the single-cell movies.

#### Aggregation Threshold Estimation

Aggregation threshold for WT and A53T α-synuclein strains was precisely quantified by training a simple classifier on the single-cell datasets. The aggregation classifier is mathematically defined by the map:

f:x∈R+→y∈{n'oaggregation',a'ggregation'}

which assigns a class label y to a normalized fluorescence level x. The map f is described as follows:

f(x)={n'oaggregation',|x<la'ggregation',|x≥l

where l is the threshold level. Thus, the classifier is characterized by the parameter l. The performance of the classifier was evaluated according to its accuracy ACC, defined as the rate of true predictions to total number of instances N:

$$ACC=\frac{TP+TN}{N}$$

where TP and TN are respectively the *true positives* and the *true negatives*. Threshold levels for both WT and A53T α-synuclein strains were quantified by solving a maximization problem (Table S4). In particular, we searched the threshold level l which maximized the prediction accuracy for WT and A53T single-cell datasets. The global optimization was performed using the MATLAB function *ga* of the *Global Optimization Toolbox*. Classifier prediction was validated by comparing its accuracy performance with the one obtained by a random classifier (Table S4). For each α-synuclein strain, random accuracy was computed using a bootstrapping procedure: 10,000 resampled datasets were generated, by randomly perturbing the class label assigned to each fluorescence level, and then classified by the classifier. The random accuracy was quantified as mean accuracy of the distribution coming from the bootstrapping procedure.

#### Single-Cell Analysis of Inclusions’ Clearance

Single-cell data were collected from the time-lapse clearance experiments (Figure 6) according to the following procedure. The custom segmentation and tracking algorithm (Method Details) processed the phase contrast images to extract single-cell traces. We visually identified the cells that showed α-synuclein-GFP inclusions during the initial calibration phase of 5 min at the beginning of the experiment when cells are still in the presence of galactose. The parameters *time-to-first-bud*, *mean fluorescence*, *cell cycle duration*, and *drop at division* (Figure 6) were measured in each cell by using the following procedures. The time-to-first-bud was computed by inspecting manually single-cell movies. The mean fluorescence was computed as the average α-synuclein-GFP fluorescence from the beginning of the experiment until the time-to-first-bud. The cell cycle duration, as well as the budded phase, were measured by inspecting manually single-cell movies. The drop at division was defined as the percentage decrease in fluorescence during the budded phase of cell cycle in each cell.

#### Mathematical Model of Fluorescence Drop at Division

Cell cycle in yeast *S. cerevisiae* is divided in unbudded phase (G1) and budded phase (S-G2-M). Cell division occurs at the end of the budded phase, when a new cell is born. To derive a mathematical model of the fluorescence drop at division, we considered the linear relationship between the size of the new-born cell ($S_{birth}^{'}$) and the size of the mother cell at the beginning of the budded phase $\left(S_{G1/S}\right)$ (Jonas et al., 2018):

$$S_{birth}^{'}=0.61\;S_{G1/S}\;.$$

We assumed that mother cell mass was constant during the budded phase (Charvin et al., 2009):

$$S_{S-G2-M}=S_{G1/S}\;.$$

Thus, the total size of the cell and its bud at division $\left(S_{div}\right)$ was:

$$S_{div}=S_{S-G2-M}+S_{birth}^{'}=0.61\;S_{G1/S}+S_{G1/S}=1.61\;S_{G1/S}\;.$$

Hence, the percentage of mass lost by the mother cell at division $\left(\Delta S_{\%}\right)$ was equal to:

$$\Delta S_{\%}=\frac{S_{div}-S_{S-G2-M}}{S_{div}}\times 100=\frac{0.61\;S_{G1/S}}{1.61\;S_{G1/S}}\times 100\approx 38\%\;.$$

We thus assumed a drop of 38% of fluorescence at each cell division.

#### Model Prediction Error of Fluorescence Drop at Division

Model prediction error of fluorescence at division was computed as percentage difference between the experimental fluorescence measured at division and the value obtained *in silico* considering the mathematical model of fluorescence drop.

#### Correlation Analysis between Time-to-First-Bud and Inclusions’ Clearance Time

Correlation analysis in Figure S2B was performed using the function *cor.test* of the R environment that computes the Pearson’s product moment correlation coefficient r. Moreover, the function also performed a test for assessing the statistical significance of the correlation analysis $\left(p-value<2.2\times 10^{-16}\right)$.

### Data and Software Availability

All the data, the computational software, and the supplemental movies generated in this study are deposited at Zenodo (https://doi.org/10.5281/zenodo.2546466).

## Supporting Citations

The following references appear in the Supplemental Information: Gietz and Sugino, 1988, Janke et al., 2004, Taxis and Knop, 2006.

## Supplemental Information

Document S1. Figures S1–S3 and Table S1–S4

Document S2. Article plus Supplemental Information
