# Supplementary material for: Quantitative Characterization of α-Synuclein Aggregation in Living Cells through Automated Microfluidics Feedback Control
Source: Cell Rep. 2019 Apr 16;27(3):916–927.e5. doi: 10.1016/j.celrep.2019.03.081 (PMC6484782; doi:10.1016/j.celrep.2019.03.081)
Supplement: Document S1. Figures S1–S3 and Table S1–S4 [file mmc1.pdf]

Cell Reports, Volume *27*

Supplemental Information

Quantitative Characterization of α-Synuclein Aggregation in Living Cells through Automated Microfluidics Feedback Control

Giansimone Perrino, Cathal Wilson, Marco Santorelli, and Diego di Bernardo

Table S1. Related to STAR Methods. Yeast strains used in this study.

Strain	Description	Genotype
yDdB001	mCherry	Mat a; TEF2pr-mCherry::URA3, his3Δ1, leu2Δ0
yDdB004	Multiple copy WT α-synuclein	Mat a; TEF2pr-mCherry::URA3, YMR082CΔ::GAL1p-SNCA-GFP-KanMX, YFR054CΔ::GAL1p-SNCA-GFP-HphMX, YCplac111-GAL1p-SNCA-GFP-LEU2, his3Δ1, leu2Δ0
yDdB005	Multiple copy A53T α-synuclein	Mat a; TEF2pr-mCherry::URA3, YMR082CΔ::GAL1p-sncaA53T-GFP-KanMX, YFR054CΔ::GAL1p-sncaA53T-GFP-HphMX, YCplac111-GAL1p-sncaA53T-GFP-LEU2, his3Δ1, leu2Δ0
yDdB023	Multiple copy A53T α-synuclein Δpdr5	Mat a; TEF2pr-mCherry::URA3, YMR082CΔ::GAL1p-sncaA53T-GFP-KanMX, YFR054CΔ::GAL1p-sncaA53T-GFP-HphMX, YCplac111-GAL1p-sncaA53T-GFP-LEU2, pdr5Δ::natNT2, his3Δ1, leu2Δ0

Table S2. Related to STAR Methods. Plasmids used in this study.

**Plasmid**	**Type**	**Source**
pYM25	Integration	Euroscarf; (Janke et al., 2004)
pYM27	Integration	Euroscarf; (Janke et al., 2004)
pRS41N	Centromeric	Euroscarf; (Taxis and Knop, 2006)
YCplac111	Centromeric	(Gietz and Sugino, 1988)
pRS304-αSynWT-GFP	Integration	(Outeiro and Lindquist, 2003)
pYM25-GALp-αSynWT-GFP	Integration	This study
pYM27-GALp-αSynWT-GFP	Integration	This study
pRS304-αSynA53T-GFP	Integration	(Outeiro and Lindquist, 2003)
pYM25-GALp-αSynA53T-GFP	Integration	This study
pYM27-GALp-αSynA53T-GFP	Integration	This study
YCplac111-GALp-αSynWT-GFP	Centromeric	This study
YCplac111-GALp-αSynA53T-GFP	Centromeric	This study

Table S3. Related to STAR Methods. Parameters for the dynamical model used by MPC.

**Parameter**	**Value**
a_{11}	0.9917
a_{12}	0.0114
a_{21}	-0.0489
a_{22}	0.9409
b_1	0.0004
b_2	-0.0117
c_1	-9.4740
c_2	-0.0113

Table S4. Related to STAR Methods. Classifier performance and validation.

**α-Synuclein**	**Threshold level**	ACC	ACC_{random}
WT	13.096	83.26	50.31 ± 0.42
A53T	7.381	85.97	51.24 ± 0.35

Threshold levels are reported in normalised fluorescence units. Accuracies are reported in percentages. Random accuracies are reported in percentages as means ± standard deviations.

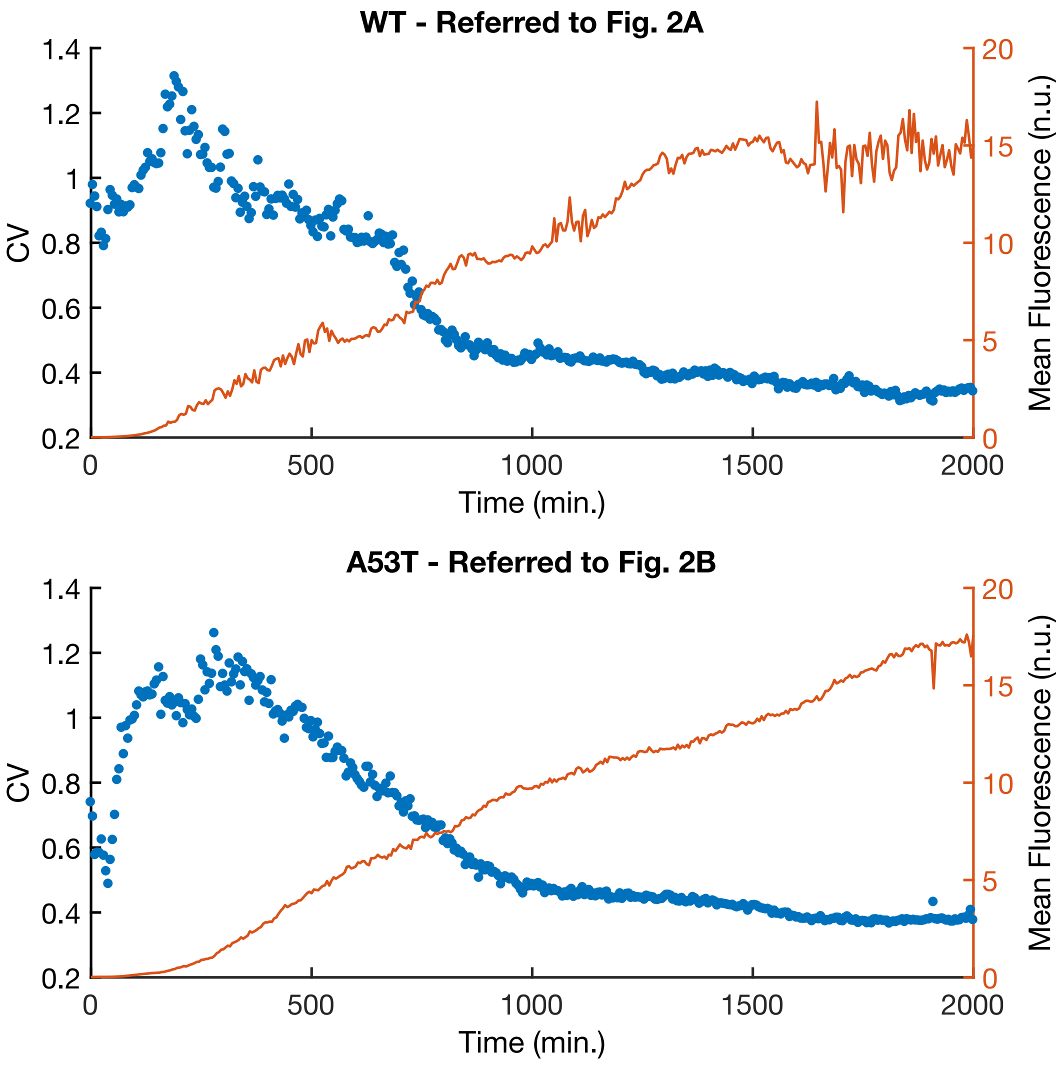

Figure S1. Related to Figure 2A and Figure 2B. Cell-to-cell variability measured during α-synuclein overexpression following galactose administration at time 0. α-Synuclein-GFP fluorescence was quantified in each cell at each time point and normalised to the red fluorescence (mCherry protein) with a custom-made image processing algorithm (STAR Methods). Solid orange lines represent population-averaged α-synuclein-GFP fluorescence as reported in **Figures 2A** and **2B**. The Coefficient of Variation (CV, blue dots) at each time point is computed as the standard deviation of fluorescence level in single cells divided by the population-averaged value of fluorescence. The CV is inversely correlated with protein expression levels.

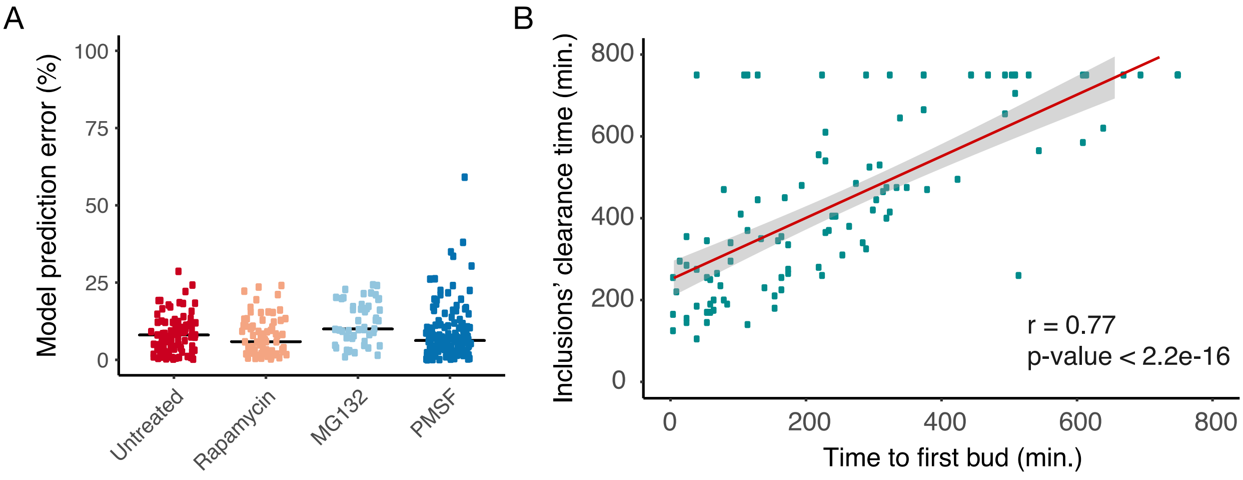

Figure S2. Related to Figure 6. Validation of A53T α-synuclein inclusion clearance dynamics.

(**A**) *In silico* model of fluorescence drop at division across single cells for the indicated conditions. Each point is the difference between the measured fluorescence at division and the one obtained according to a mathematical model of dilution assuming a drop of 38% of fluorescence for each cell cycle (STAR Methods). As each cell undergoes multiple divisions during the experiment, the number of points does not correspond to the number of cells: untreated (n = 91 points from 25 cells), rapamycin (n = 73 points from 14 cells), MG132 (n = 49 points from 24 cells), and PMSF (n = 137 points from 48 cells). Solid black lines are the medians of the distribution in each condition.

(**B**) *Time-to-first-bud* and *inclusions' clearance time* are correlated in individual cells across conditions (Pearson's product moment correlation coefficient $r = 0.77$, $p - value < 2.2e - 16$; STAR Methods). The *time-to-first-bud (TFB)* of a cell is defined as the time elapsed between the beginning of the experiment, when α-synuclein expression is inhibited, and the formation of the first cell bud. The inclusions' clearance time of a cell is defined as the time it takes for α-synuclein inclusions to disappear. The linear regression line is reported (red line), as well as its confidence interval (shaded grey area) (STAR Methods). Single-cell data were collected from the experiments shown in **Figure 6**. The number of points corresponds to the total number of cells across conditions and it is equal to 25 (untreated) + 14 (rapamycin) + 24 (MG132) + 48 (PMSF) = 111.

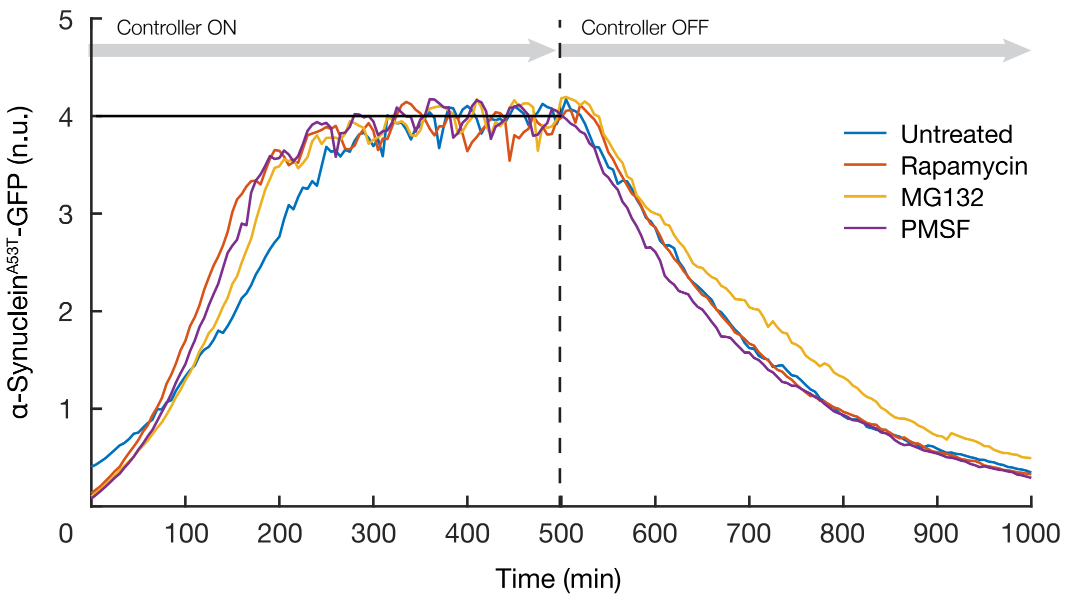

Figure S3. Related to Figure 6. Effects of proteasome and autophagy modulation on A53T α-synuclein clearance dynamics below the aggregation threshold. Time-lapse microfluidics control experiments in the mutant Δpdr5 A53T strain across the indicated conditions. α-Synuclein expression level was regulated to 4 normalised units for 500 min, that is below the aggregation threshold, followed by inhibition of α-synuclein expression for additional 500 min by a Model Predictive Control strategy (STAR Methods). α-Synuclein-GFP fluorescence was quantified in each cell and normalised to the red fluorescence (mCherry protein) with a custom-made image processing algorithm (STAR Methods). At the population level, treatment either with rapamycin (red line) or PMSF (purple line) had no measurable effects on clearence dynamics. Cells treated with MG132 (yellow line) showed a marginal impairment of the clearance dynamics likely driven by its effects on the cell cycle and hence dilution, rather than to a direct effect of proteasome inhibition on α-synuclein. Analysis of single cell traces could not be performed in this condition as in the absence of inclusions, yeast cells continuosly proliferate and after a short time they are pushed out of the field of view by new cells being born.
